# Fibromyalgia neurobehavioral phenotypes remain distinct across varying levels of pharmacologic exposure: evidence from a multisite cohort (*n* = 695)

**DOI:** 10.1017/S1092852926101199

**Published:** 2026-09-22

**Authors:** Kevin V. Hackshaw, Michelle M. Osuna-Diaz, Katherine R. Sebastian, Shreya Madhav Nuguri, Silvia deLamo Castellvi, Lianbo Yu, M. Monica Giusti, Zhanna Mikulik, Luis Rodriguez-Saona

**Affiliations:** 1 Division of Rheumatology, Department of Medicine, The University of Texas at Austin Dell Medical School, Austin, USA; 2 Department of Medicine, The University of Texas at Austin Dell Medical School, USA; 3 Food Sciences and Technology, The Ohio State University, USA; 4 Bioinformatics, The Ohio State University Wexner Medical Center, USA; 5 Division of Immunology and Rheumatology, Department of Medicine, The Ohio State University Wexner Medical Center, Columbus, USA

**Keywords:** Fibromyalgia, central sensitization, neurobehavioral phenotypes, affective symptoms, chronic pain, CNS disorders

## Abstract

**Objective:**

To determine whether pharmacologic exposure is associated with differences in fibromyalgia neurobehavioral phenotypes and whether medication burden explains symptom variability within phenotypes.

**Methods:**

We analyzed a multisite cohort of 695 adults meeting fibromyalgia criteria, stratified into low- and high-impact phenotypes using a disability-based classification. Medication burden was quantified as total, centrally acting, and noncentrally acting medication counts. Between-phenotype differences and within-phenotype associations between medication burden and symptom severity were evaluated using nonparametric tests, Spearman correlation analyses, and multivariable linear regression models. Phenotypes were interpreted within the framework of centrally mediated neurobehavioral dysfunction.

**Results:**

Participants classified within the high-impact neurobehavioral phenotype (*n* = 175) demonstrated substantially greater symptom burden across fibromyalgia impact, central sensitization, depressive symptoms, pain severity, and vitality compared with low-impact participants (*n* = 520). Centrally acting medication burden increased stepwise with phenotype severity, whereas noncentrally acting medication use did not differ between phenotypes. Within phenotypes, centrally acting medication burden showed weak and nonsignificant associations with symptom severity after adjustment for demographic covariates, explaining minimal variance in fibromyalgia impact (*R* ^2^ < 0.05).

**Conclusions:**

Fibromyalgia symptom phenotypes remained distinct across varying levels of pharmacologic exposure, and medication burden explained little within-phenotype symptom variability. Medication burden primarily reflects downstream clinical response to symptom severity rather than a determinant of phenotype organization. These findings support the use of stable multidimensional phenotypes for mechanistic, neurobiological, and stratified intervention studies in chronic pain and related central nervous system disorders.

## Introduction

Fibromyalgia is a centrally mediated chronic pain disorder characterized by widespread pain, fatigue, cognitive dysfunction, affective symptoms, and impaired physical and social functioning.^1^ ^–^^5^ Despite its high prevalence and clinical impact, fibromyalgia remains diagnostically and therapeutically challenging, in part due to substantial interindividual heterogeneity in symptom severity and disease burden.^6^ ^–^^10^ This heterogeneity has complicated efforts to identify mechanistic subgroups, predict outcomes, and tailor treatment strategies. Increasing evidence suggests that fibromyalgia symptoms may organize into reproducible multidimensional patterns involving sensory, affective, and functional domains.

Data-driven phenotyping studies increasingly suggest that fibromyalgia symptoms organize along dominant severity or impact dimensions rather than discrete symptom clusters.^11^ ^–^^15^ More recently, our multisite investigations have demonstrated that these phenotypes are highly reproducible across independent cohorts, remain consistent despite pharmacologic exposure, and extend beyond traditional disease labels into related chronic multisystem conditions.^16^ ^–^^20^ Collectively, these observations support the concept that multidimensional symptom phenotypes represent reproducible organizational features of fibromyalgia rather than artifacts of medication use or individual cohort characteristics. Disability-based stratification approaches have proven clinically intuitive and reproducible, capturing meaningful differences in global symptom burden, functional impairment, and quality of life.^21^ ^,^^22^ However, the relationship between these phenotypes and medication exposure remains unclear. Centrally acting medications—including antidepressants, gabapentinoids, muscle relaxants, and centrally acting analgesics—are commonly prescribed in fibromyalgia, yet whether medication burden meaningfully contributes to phenotypic differentiation or simply reflects clinical response to severity has not been systematically examined.

Although previous investigations have demonstrated reproducible fibromyalgia symptom phenotypes and numerous studies have described medication use in fibromyalgia, whether pharmacologic exposure itself substantially influences phenotype assignment or instead reflects downstream treatment of disease severity has not been systematically examined. Resolving this question is important because medication burden could potentially confound phenotype-based biomarker studies, mechanistic investigations, and stratified therapeutic trials.

Clarifying this relationship has important implications. If medication burden defines phenotypes, pharmacologic exposure could confound phenotyping efforts. Conversely, if medication use primarily reflects downstream response to disease impact, it should be interpreted as a contextual clinical marker rather than a driver of heterogeneity. In this study, we examined medication burden across disability-defined fibromyalgia phenotypes to determine whether centrally acting and noncentrally acting medications differentially characterize phenotype structure. From a neuropsychiatric and central nervous system (CNS) perspective, clarifying whether treatment exposure shapes or merely reflects symptom organization is critical for interpreting phenotypic structure in centrally mediated neurobehavioral disorders. This question is particularly relevant across centrally mediated disorders in which treatment exposure and symptom expression are tightly interrelated and difficult to disentangle.

## Methods

### Participants and study setting

Adults aged 18 to 80 years meeting the 2016 American College of Rheumatology (ACR) fibromyalgia criteria were recruited from two academic medical centers: The University of Texas at Austin and The Ohio State University.^1^ ^,^^7^ ^,^^23^ Recruitment occurred from September 2020 to October 2025 as part of an ongoing biomarker and phenotyping study. All participants provided written informed consent, and institutional review board approval was obtained at both sites (supervising IRB: UT Austin IRB #2020030008).

### Study population

Participants were drawn from a clinical cohort of adults meeting the fibromyalgia criteria and had completed standardized assessments of symptom severity, functional impact, and medication use.^24^ ^–^^29^ Individuals without sufficient data to derive disability-based phenotype classification were excluded from phenotype-stratified analyses.

### Phenotype classification

Participants were stratified using a prespecified disability-based classification derived from the Revised Fibromyalgia Impact Questionnaire (FIQR). High-impact fibromyalgia was defined as an FIQR score ≥62, corresponding to the 75th percentile of the cohort distribution, whereas participants with FIQR scores <62 were classified as low impact. This classification yielded 175 participants in the high-impact group and 520 participants in the low-impact group. The threshold was established as part of our previously developed disability-based stratification framework and was not optimized post hoc in the present study. Participants meeting or exceeding the threshold (FIQR score ≥62) were assigned to the high-impact phenotype, whereas those with scores below 62 were assigned to the low-impact phenotype. This pragmatic threshold was selected to identify participants with the greatest functional impairment while preserving clinically interpretable subgroup sizes and maintaining consistency with our previously published multisite and longitudinal phenotyping framework.^16^ ^,^^19^ This approach was selected for clinical interpretability and alignment with real-world assessment of disease burden.^30^ ^–^^32^

### Clinical measures

Symptom severity was assessed using validated patient-reported measures of fibromyalgia impact, central sensitization, depressive symptoms, pain severity, and vitality.^24^ ^–^^29^ Demographic variables included age, sex, and body mass index (BMI). The selected symptom domains were intended to capture multidimensional neurobehavioral functioning relevant to central sensitization and affective symptom burden.

### Medication burden

Medication use was categorized into centrally acting and noncentrally acting agents based on pharmacologic class. Centrally acting medications were grouped according to their predominant mechanisms of action within the CNS rather than therapeutic indication. These included antidepressants, gabapentinoids, skeletal muscle relaxants, centrally acting analgesics, benzodiazepines, and related psychotropic medications because each has recognized effects on pain modulation, affective processing, or sensory amplification. Medication burden was quantified as total medication count, centrally acting medication count, and noncentrally acting medication count at baseline.

### Statistical analysis

Continuous variables are presented as mean ± standard deviation (SD), and categorical variables as frequencies and percentages. Baseline demographic, clinical, and medication characteristics were compared between the low-impact and high-impact fibromyalgia phenotypes using Mann–Whitney *U* tests for continuous variables because several outcome measures demonstrated nonnormal distributions, and χ^2^ tests were used for categorical variables. For the family of continuous between-phenotype comparisons, *P* values were adjusted for multiple testing using the Benjamini–Hochberg false discovery rate (FDR) procedure; both raw and FDR-adjusted *P* values are reported in the Supplementary Material.

Medication burden was quantified as the total number of medications, centrally acting medication count, and noncentrally acting medication count recorded at the baseline study visit. Centrally acting medications included antidepressants (tricyclic antidepressants, serotonin–norepinephrine reuptake inhibitors, selective serotonin reuptake inhibitors), gabapentinoids, benzodiazepines, skeletal muscle relaxants, centrally acting analgesics, and other psychotropic medications with recognized effects on central pain processing, affective regulation, or sensory modulation. Medications were classified according to their predominant mechanism of action within the CNS rather than therapeutic indication.

Associations between medication burden and clinical outcome measures within each phenotype were initially evaluated using Spearman rank correlation coefficients (ρ) because medication counts and several symptom variables exhibited nonparametric distributions. Correlation coefficients were interpreted according to conventional thresholds (weak, <0.30; moderate, 0.30–0.49; strong, ≥0.50).

To determine whether medication burden independently contributed to symptom severity after adjustment for potential confounding variables, separate multivariable linear regression models were constructed for each phenotype. The primary dependent variable was the FIQR score. Secondary models evaluated Central Sensitization Inventory (CSI), Beck Depression Inventory (BDI), McGill Pain Questionnaire (MPQ), and SF-36 Vitality scores. Independent variables included centrally acting medication count together with age, sex, BMI, and study site as covariates. Regression assumptions were evaluated by inspection of residual plots, normality of residuals, homoscedasticity, and variance inflation factors to assess multicollinearity. Representative regression diagnostic plots are shown in Supplementary Figure S1.

For each regression model, standardized regression coefficients (β), standard error (SE), 95% confidence interval (CI), two-sided *P* values, and adjusted coefficients of determination (adjusted *R* ^2^) were calculated to quantify both statistical significance and the magnitude of association. Statistical significance was defined as a two-sided *P* value <0.05. Because regression analyses are considered hypothesis driven and exploratory with predefined outcomes, effect sizes and confidence intervals were emphasized over sole reliance on statistical significance. The disability-based phenotype assignment was prespecified before regression modeling and remained fixed throughout all subsequent analyses.

Complete regression coefficients, Spearman correlation matrices, model diagnostics, and medication classification details are provided in Supplementary Tables S1–S4 to enhance transparency while maintaining the readability of the primary manuscript. Patterns of missing data are summarized in Supplementary Figure S3.

All statistical analyses were performed using R version 4.5.2 (R Foundation for Statistical Computing, Vienna, Austria).^33^

Exclusion criteria included active inflammatory rheumatic disease, uncontrolled psychiatric illness, untreated thyroid disease, active infection, pregnancy, or inability to complete study assessments.

## Results

### Cohort characteristics

Complete regression coefficients, correlation matrices, and model diagnostics are presented in Supplementary Tables S1–S4; the principal findings are summarized in the following sections. Among 695 participants with complete phenotype data, 520 were classified as low impact and 175 as high impact. The high-impact neurobehavioral phenotype exhibited a greater proportion of female participants and higher BMI, while age distributions were similar across groups (Table 1).

**Table 1.** Demographic, clinical, and medication characteristics of fibromyalgia phenotypes identified by disability-based stratification Table 1. long description.

Variable	High Impact (*n* = 175)	Low Impact (*n* = 520)
Female (%)	96.6	86.3
Age (years)	45.1 ± 12.1	45.2 ± 15.6
BMI (kg/m^2^)	34.5 ± 10.1	30.6 ± 8.3
FIQR	74.2 ± 8.7	31.3 ± 20.9
CSI	72.2 ± 12.0	50.7 ± 17.8
BDI	27.2 ± 10.2	14.4 ± 9.2
MPQ	133.4 ± 42.8	66.4 ± 41.2
Centrally acting medications (CAM) (*n*)	1.3 ± 1.1	0.8 ± 1.0

### Symptom severity across phenotypes

The high-impact phenotype exhibited markedly higher fibromyalgia impact scores, central sensitization, depressive symptom burden, and pain severity, along with lower vitality scores compared with the low-impact phenotype (Table 1). These findings demonstrate clear differences between the disability-defined fibromyalgia groups.

### Medication burden across phenotypes

**Figure 1.**
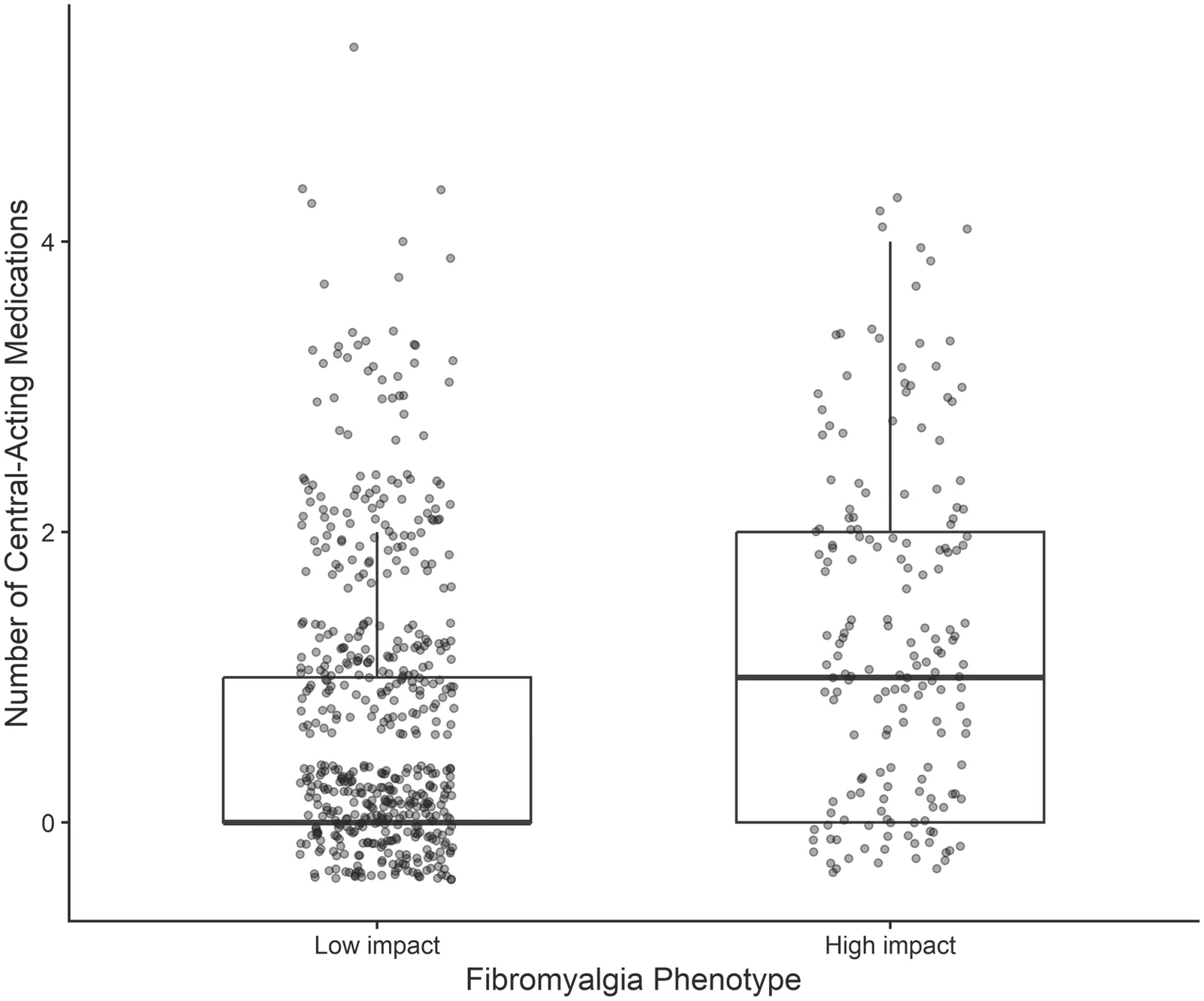
Distribution of centrally acting medication burden by fibromyalgia impact group. Figure 1 long description.

**Figure 2.**
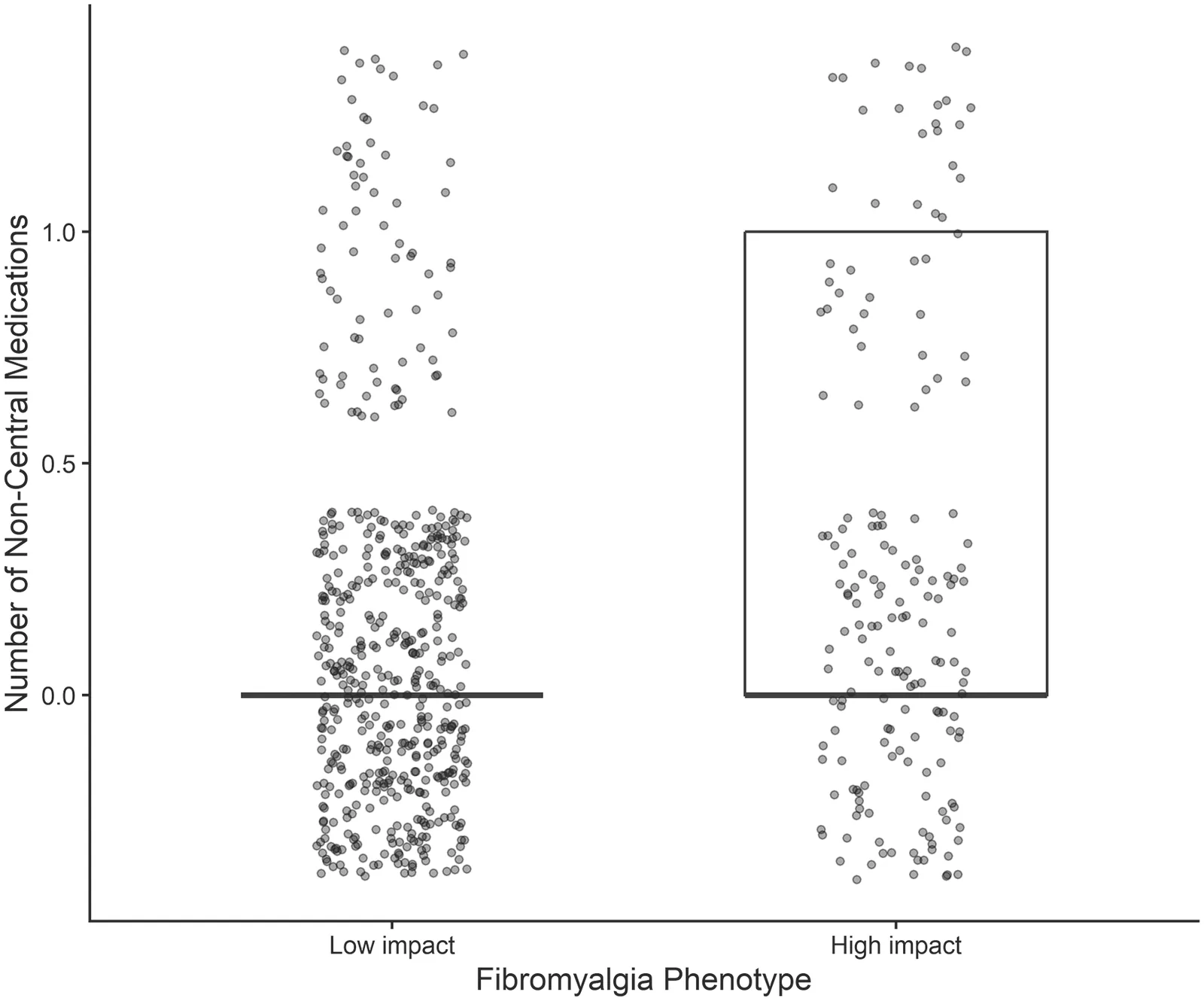
Multidimensional clinical symptom profiles in low- and high-impact fibromyalgia. Figure 2 long description.

Total medication burden was significantly higher in the high-impact phenotype. This difference was driven specifically by centrally acting medications, which demonstrated a clear stepwise increase with phenotype severity (Figure 1). In contrast, noncentrally acting medication use did not differ between the low- and high-impact phenotypes (Figure 2). Standardized effect sizes and complete regression coefficients are presented in Supplementary Tables S1–S3. Relationships among symptom domains and medication burden are illustrated in Supplementary Figure S4. Standardized regression coefficients are summarized visually in Supplementary Figure S8.

### Within-phenotype associations

Within phenotypes, centrally acting medication burden demonstrated weak correlations with symptom severity. After multivariable adjustment, these associations were attenuated and accounted for only a small proportion of the observed variability (adjusted *R* ^2^ consistently <0.05).

Supplementary Figures S5–S8 include a forest plot of multivariable linear regression models, the clinical profile of high- versus low-impact fibromyalgia groups, the distribution of medication burden disability groups, and a summary of regression effects, respectively.

## Discussion

In this large multisite fibromyalgia cohort, we extend a growing body of work from our group demonstrating reproducible fibromyalgia phenotypes across cross-sectional cohorts, longitudinal follow-up, medication exposure, and related chronic multisystem disorders by examining whether medication burden itself alters phenotype organization or explains symptom severity within established phenotypes.^16^ ^–^^20^ Our findings demonstrate that centrally acting medication use differs across clinically defined fibromyalgia phenotypes; however, medication burden explains little symptom variability within individual phenotypes. Together, these findings provide population-level evidence that fibromyalgia symptom phenotypes remain robust despite pharmacologic exposure and are consistent with relatively reproducible symptom organization rather than associated with treatment exposure configurations. These findings are consistent with current models conceptualizing fibromyalgia as a centrally mediated disorder characterized by reproducible co-occurrence of sensory, affective, and functional symptom dimensions.

Consistent with our prior clustering-based stratification analyses, pragmatic multidimensional clustering identified low- and high-impact fibromyalgia phenotypes with graded differences in global impact, central sensitization, mood disturbance, pain, and functional impairment. Importantly, demographic characteristics were similar across phenotypes, reinforcing that observed differences were not attributable to age, sex, or body composition. Against this background of consistently observed phenotype structure, centrally acting medication burden demonstrated a clear and stepwise increase from lower- to higher-impact phenotypes, whereas noncentrally acting medication use did not differ across phenotypes. This specificity strongly suggests that treatment escalation in fibromyalgia preferentially targets CNS-directed symptoms rather than reflecting generalized medication exposure or comorbidity burden. This dissociation between treatment intensity and symptom structure is consistent with the possibility that treatment escalation may have limited capacity to modify underlying phenotype architecture.

However, when examined within phenotypes, centrally acting medication burden explained little variance in symptom severity. Unadjusted analyses demonstrated only weak associations between central medication count and global fibromyalgia impact, and these associations were small in magnitude and largely confined to FIQR scores. Critically, after adjustment for age, sex, and BMI, central medication burden was no longer independently associated with symptom severity in any phenotype. Pain intensity, central sensitization, fatigue, and depressive symptoms were not meaningfully associated with medication burden within phenotypes. These findings argue against a simple dose–response relationship between polypharmacy and symptom severity and suggest that escalating medication regimens do not fundamentally reshape symptom expression once phenotype membership is established.

These results have important clinical and conceptual implications. In routine practice, fibromyalgia management often involves sequential addition of centrally acting agents in response to persistent symptoms, with the implicit assumption that greater pharmacologic intensity will translate into proportional symptom improvement. Our data challenge this assumption by demonstrating that, within phenotypes, medication burden confers minimal explanatory power for symptom severity. Instead, phenotype membership itself appears to capture the dominant structure of symptom burden. This distinction helps reconcile two commonly observed clinical realities: first, that patients with more severe fibromyalgia are more likely to be treated with multiple centrally acting medications, and second, that such treatment escalation frequently yields diminishing returns.^34^ ^–^^36^ From a pathophysiologic perspective, these findings support models in which fibromyalgia represents a disorder of reproducible central processing phenotypes rather than a continuously modifiable pharmacologic gradient.

The present findings may also have broader implications for understanding centrally mediated neuropsychiatric disorders characterized by robust symptom architectures despite substantial treatment exposure. These observations also align with our recent conceptual work proposing that central sensitization disorders may represent broader neurobehavioral states characterized by reproducible symptom organization across multiple clinical conditions rather than isolated disease entities.^37^ In fibromyalgia, centrally acting pharmacotherapies are frequently prescribed to target pain amplification, affective symptoms, sleep disturbance, and sensory dysregulation. However, the persistence of consistently observed multidimensional phenotypes despite pharmacologic escalation suggests that underlying neurobehavioral organization may be only partially modifiable using currently available therapies. This framework aligns with emerging models emphasizing altered CNS processing, affective abnormalities, and persistent central sensitization as core organizing features of fibromyalgia. The present study represents the next step in an evolving program of research examining biological and clinical phenotyping in fibromyalgia. Previous investigations demonstrated that data-driven fibromyalgia phenotypes are reproducible across independent academic centers, remain stable over repeated clinical assessments, and are preserved despite differences in pharmacologic exposure. More recently, we reported that similar multidimensional symptom profiles extend beyond fibromyalgia into long COVID and that depression, pain amplification, and functional impairment demonstrate comparable interrelationships across fibromyalgia, rheumatoid arthritis, and systemic lupus erythematosus. The current analysis builds directly upon these observations by demonstrating that medication burden itself contributes little to symptom variability within established phenotypes, suggesting that pharmacologic exposure functions primarily as a marker of disease severity rather than a determinant of phenotype organization.^16^ ^–^^20^ Unlike previous studies, the present analysis specifically examined whether medication burden independently contributes to phenotype differentiation after accounting for demographic covariates. The findings suggest that medication exposure functions primarily as a marker of disease severity rather than a determinant of phenotype assignment. Whereas our earlier analyses demonstrated that medication exposure does not alter phenotype structure, the current findings clarify that medication burden also does not meaningfully explain within-phenotype severity. Together, these observations reinforce the concept that fibromyalgia phenotypes reflect underlying neurobiological and psychosocial processes that are only partially responsive to available pharmacologic therapies. This framework aligns with emerging biomarker, neuroimaging, and systems-level studies suggesting that central sensitization, affective processing, and functional impairment are tightly coupled within individuals and resistant to simple pharmacologic modulation.^37^ ^–^^39^

Several strengths of this study merit emphasis. The analysis leverages a large, well-characterized fibromyalgia cohort drawn from two academic centers, enhancing generalizability while preserving methodological rigor. Medication burden was derived from detailed medication lists and classified using a priori definitions that distinguished centrally acting from noncentrally acting agents, allowing for mechanistic specificity. The availability of detailed medication information allowed direct evaluation of medication burden as a potential source of phenotypic confounding, an aspect that has received relatively little attention in previous fibromyalgia phenotyping studies. Phenotypes were identified using disability-based classification threshold based on validated symptom and functional measures, minimizing investigator bias and avoiding reliance on single-domain severity scales. Importantly, the analytic strategy explicitly separated between-phenotype and within-phenotype effects, reducing the risk of confounding by indication and enabling a more nuanced interpretation of medication–severity relationships.

Several limitations should also be acknowledged. Residual confounding by indication cannot be excluded because patients with more severe symptoms are more likely to receive multiple centrally acting medications. Additional sources of unmeasured confounding, including socioeconomic status, education level, healthcare access, treatment adherence, disease duration, and prescribing practices between study sites, may also have influenced medication burden. Because medication exposure and symptom severity were measured contemporaneously, the cross-sectional design precludes inference regarding temporal or causal relationships between pharmacologic treatment and phenotype organization.

Medication exposure was assessed cross-sectionally and did not capture dose, duration, adherence, or prior treatment failures, which may influence symptom trajectories over time. Although the pragmatic clustering approach allowed inclusion of a large proportion of the cohort, some participants were excluded due to incomplete symptom data, and clustering relied on baseline assessments rather than longitudinal trajectories. Additionally, while centrally acting medications were grouped based on mechanistic class, individual agents within classes may exert heterogeneous effects that were not examined separately. Finally, as an observational study, causal inferences regarding the effects of medication burden on symptom severity cannot be made.

Despite these limitations, the present findings have direct relevance for both clinical practice and future research. The dissociation between medication burden and within-phenotype severity underscores the need for phenotype-informed treatment strategies that extend beyond pharmacologic escalation. These results support a shift toward integrative, multimodal approaches that target phenotype-specific mechanisms, including behavioral, rehabilitative, and neuromodulatory interventions.^40^ ^–^^43^ From a research perspective, reproducible phenotypes that are not substantially distorted by medication burden provide a robust framework for mechanistic studies, biomarker discovery, and clinical trial enrichment.^6^ ^,^^44^ ^–^^48^ These findings have broader implications for understanding symptom organization across heterogeneous centrally mediated disorders, where disentangling intrinsic symptom structure from treatment effects remains a central challenge across heterogeneous conditions. These results support the use of phenotype-based stratification frameworks that remain valid in real-world, treatment-exposed populations.

In conclusion, centrally acting medication burden differed across clinically defined fibromyalgia phenotypes but explained little symptom variability within individual phenotypes after adjustment for demographic covariates. These findings suggest that multidimensional symptom phenotypes remain distinguishable despite variation in pharmacologic treatment and support continued investigation of phenotype-based approaches for mechanistic studies and precision therapeutics.

## Supporting information

10.1017/S1092852926101199.sm001 Hackshaw et al. supplementary material 1 Hackshaw et al. supplementary material

10.1017/S1092852926101199.sm002 Hackshaw et al. supplementary material 2 Hackshaw et al. supplementary material

## Data Availability

The data supporting the findings of this study contain protected health information and cannot be made publicly available because of institutional review board (IRB) restrictions. Deidentified data may be made available from the corresponding author upon reasonable request, contingent upon IRB approval and execution of an appropriate data use agreement. All statistical analyses were conducted using R version 4.5.2.

## Supplementary material

The supplementary material for this article can be found at http://doi.org/10.1017/S1092852926101199.

## AI statement

Portions of manuscript preparation were assisted using OpenAI ChatGPT (GPT-5.5; OpenAI, accessed June–July 2026 via https://chatgpt.com). The tool was used to assist with editorial refinement of manuscript text, language editing, code formatting, and graphical layout of selected Supplementary Figures. AI tools were not used to generate, analyze, or interpret study data. All statistical analyses, study design, interpretation of results, figure content, and scientific conclusions were independently performed, verified, and approved by all of the authors. All AI-assisted material was critically reviewed, edited, and validated by all of the authors prior to submission. The authors accept full responsibility for the accuracy, integrity, and content of the manuscript.

## Long descriptions

### Table 1. Long description

Baseline demographic and clinical characteristics by fibromyalgia impact group. Participants were stratified into low- and high-impact fibromyalgia groups using a disability-based classification threshold corresponding to the 75th percentile of functional impact. High-impact phenotype defined as FIQR ≥62 (75th percentile of the cohort distribution); low-impact phenotype defined as FIQR <62. Demographic characteristics are shown in the upper panel and clinical symptom severity and medication burden in the lower panel. Continuous variables are presented as mean ± standard deviation and categorical variables as percentage. FIQR, Fibromyalgia Impact Questionnaire–Revised; CSI, Central Sensitization Inventory; BDI, Beck Depression Inventory; MPQ, McGill Pain Questionnaire; CAM, centrally acting medications.

Navigate back to Table 1..

### Figure 1 Long description

The number of centrally acting medications (CAM) reported at the study visit is shown for low- and high-impact fibromyalgia groups defined using a disability-based classification threshold corresponding to the 75th percentile of functional impact. Distributions illustrate increasing medication burden with higher impact status, demonstrating greater centrally acting medication burden among patients with the high-impact phenotype.

Navigate back to Figure 1.

### Figure 2 Long description

Group-level differences are shown across key symptom and functional domains, including fibromyalgia impact (FIQR), central sensitization (CSI), depressive symptoms (BDI), pain burden (MPQ), and physical functioning. Patients classified as high impact demonstrate consistently greater symptom burden across domains, illustrating graded differences across symptom domains.

Navigate back to Figure 2.
