# Supplementary material for: Fibromyalgia neurobehavioral phenotypes remain distinct across varying levels of pharmacologic exposure: evidence from a multisite cohort (n = 695)
Source: CNS Spectr. 2026 Sep 22;31(1):e38. doi: 10.1017/S1092852926101199 (PMC13732593; doi:10.1017/S1092852926101199)
Supplement: Supplementary file 1 [file S1092852926101199sup001.docx]

**Supplementary Figures**

Figure layouts for the supplementary figures were prepared with assistance from OpenAI ChatGPT (GPT-5.5). All underlying data, statistical analyses, graphical outputs, scientific interpretation, and final figure content were generated, verified, and approved by the authors.

**Supplementary Figure S1**

Regression Diagnostic Assessment of the Multivariable Linear Regression Model Evaluating the Association Between Clinical Variables and SF-36 Physical Component Summary Scores

**Legends**

**Supplementary Figure S1A**

**Residuals versus fitted values for the multivariable regression model.** Residuals are randomly distributed around zero without substantial curvature, supporting the assumption of linearity.

**Supplementary Figure S1B**

**Normal quantile–quantile plot of standardized residuals.** Residuals closely follow the theoretical normal distribution with only minor deviation at the distribution tails.

**Supplementary Figure S1C**

**Scale–location plot.** The square root of the standardized residuals is plotted against fitted values to evaluate homoscedasticity. The absence of pronounced funneling indicates relatively constant residual variance across the range of fitted values.

**Supplementary Figure S1D**

**Cook's distance plot.** Each point represents an individual observation. The dashed horizontal reference line corresponds to the conventional threshold (4/n) used to identify potentially influential observations. Although several observations exceeded this threshold, no single observation demonstrated disproportionate influence sufficient to alter overall model interpretation.

**Supplementary Figure S1E**

**Leverage versus externally studentized residual plot.** This plot simultaneously evaluates observation leverage and residual magnitude. Dashed horizontal lines represent externally studentized residuals of ±3, while the vertical dashed line represents the conventional leverage threshold (2p/n). No observations demonstrated both excessive leverage and extreme residual values sufficient to substantially influence model estimates.
