# Supplementary material for: Fibromyalgia neurobehavioral phenotypes remain distinct across varying levels of pharmacologic exposure: evidence from a multisite cohort (n = 695)
Source: CNS Spectr. 2026 Sep 22;31(1):e38. doi: 10.1017/S1092852926101199 (PMC13732593; doi:10.1017/S1092852926101199)
Supplement: Supplementary file 2 [file S1092852926101199sup002.docx]

**Supplementary Table S1**

**Clinical Characteristics According to Disability Phenotype (FIQR <62 vs ≥62)**

| **Variable** | **Low Impact (n=520) Mean ± SD** | **High Impact (n=175) Mean ± SD** | **Welch t-test P** | **Mann–Whitney P** | **FDR-adjusted P** |
| --- | --- | --- | --- | --- | --- |
| Age (years) | 45.2 | 45.1 | 0.920 | 0.794 | 0.920 |
| BMI (kg/m²) | 30.6 | 34.5 | 2.0×10⁻⁵ | 9.5×10⁻⁶ | 2.1×10⁻⁵ |
| Central Sensitization Inventory | 50.7 | 72.2 | 4.4×10⁻⁵¹ | 1.5×10⁻⁴² | 2.3×10⁻⁵⁰ |
| Beck Depression Inventory | 14.4 | 27.2 | 1.5×10⁻³⁴ | 5.0×10⁻³⁵ | 4.1×10⁻³⁴ |
| McGill Pain Questionnaire | 66.4 | 133.4 | 4.7×10⁻⁴⁶ | 6.9×10⁻⁴⁴ | 1.5×10⁻⁴⁵ |
| FIQR | 31.3 | 74.2 | <10⁻¹⁶⁰ | <10⁻⁸⁶ | <10⁻¹⁶⁶ |
| SF-36 Physical Function | 49.4 | 18.9 | 6.4×10⁻⁵¹ | 1.7×10⁻³⁷ | 2.6×10⁻⁵⁰ |
| SF-36 Role Physical | 22.9 | 3.1 | 3.8×10⁻²³ | 3.7×10⁻¹³ | 6.8×10⁻²³ |
| SF-36 Bodily Pain | 42.1 | 15.8 | 2.0×10⁻⁵³ | 2.4×10⁻⁴⁰ | 1.6×10⁻⁵² |
| SF-36 General Health | 38.9 | 22.7 | 1.2×10⁻²² | 1.3×10⁻¹⁸ | 2.0×10⁻²² |
| SF-36 Vitality | 28.8 | 12.2 | 2.8×10⁻²⁴ | 4.8×10⁻²⁰ | 5.7×10⁻²⁴ |
| SF-36 Social Function | 60.9 | 32.8 | 1.2×10⁻³⁰ | 1.7×10⁻²⁸ | 2.8×10⁻³⁰ |
| SF-36 Role Emotional | 45.9 | 14.6 | 3.0×10⁻²² | 6.1×10⁻¹⁶ | 4.3×10⁻²² |
| SF-36 Emotional Well-being | 62.7 | 45.9 | 3.7×10⁻¹⁷ | 1.4×10⁻¹⁶ | 4.9×10⁻¹⁷ |
| Total Medication Burden | 0.94 | 1.57 | 3.6×10⁻⁸ | 3.2×10⁻⁹ | 4.5×10⁻⁸ |
| CNS Medication Burden | 0.80 | 1.31 | 3.1×10⁻⁷ | 3.1×10⁻⁸ | 3.6×10⁻⁷ |

**Footnote**

Disability groups were defined using an FIQR threshold of 62 derived from the original manuscript analysis workspace. P values were adjusted for multiple comparisons using the Benjamini–Hochberg false discovery rate procedure.

**Supplementary Table S1. Complete multivariable linear regression analyses evaluating the association between centrally acting medication burden and clinical outcomes within disability-defined fibromyalgia phenotypes.**

Separate multivariable linear regression models were constructed within the low-impact (FIQR <62) and high-impact (FIQR ≥62) phenotypes. Models were adjusted for age, sex, body mass index (BMI), and study site. β coefficients represent the independent association between centrally acting medication count and each outcome.

**Interpretation:** Across all adjusted models, centrally acting medication burden demonstrated statistically significant but small independent associations with clinical outcomes, accounting for less than 5% of the observed variance within disability-defined phenotypes.

**Supplementary Table S4**

**Medication Classification Used for CNS Medication Burden**

| Drug/Class | CNS Medication Burden |
| --- | --- |
| Amitriptyline | Yes |
| Nortriptyline | Yes |
| Duloxetine | Yes |
| Milnacipran | Yes |
| Venlafaxine | Yes |
| Fluoxetine | Yes |
| Gabapentin | Yes |
| Pregabalin | Yes |
| Cyclobenzaprine | Yes |
| Baclofen | Yes |
| Tizanidine | Yes |
| Carisoprodol | Yes |
| Tramadol | Yes |
| Topiramate | Yes |
| Bupropion | Yes |
| NSAIDs | No |
| Acetaminophen | No |

Footnote:

CNS medication burden represented the total number of centrally acting medications reported by each participant.

**Supplementary Tables S2-S5**

*CNS Spectrums revision*

**Supplementary Table S2. Within-phenotype Spearman correlation analyses.**

| **Phenotype** | **Variable 1** | **Variable 2** | **N** | **Spearman ρ** | **Raw P** | **BH FDR-adjusted P** |
| --- | --- | --- | --- | --- | --- | --- |
| Low | FIQR | CSI | 465 | 0.492 | 1.06e-29 | 4.94e-29 |
| Low | FIQR | BDI | 474 | 0.418 | 1.69e-21 | 4.31e-21 |
| Low | FIQR | MPQ | 457 | 0.488 | 1.09e-28 | 4.37e-28 |
| Low | FIQR | SF-36 Vitality | 451 | -0.441 | 7.61e-23 | 2.37e-22 |
| Low | FIQR | BMI | 419 | 0.015 | 0.761 | 0.789 |
| Low | FIQR | Total medication burden | 520 | 0.391 | 1.96e-20 | 4.57e-20 |
| Low | FIQR | CNS medication burden | 520 | 0.407 | 3.69e-22 | 1.03e-21 |
| Low | CSI | BDI | 462 | 0.679 | 1.05e-63 | 1.47e-62 |
| Low | CSI | MPQ | 455 | 0.628 | 2.98e-51 | 2.78e-50 |
| Low | CSI | SF-36 Vitality | 446 | -0.618 | 2.87e-48 | 2.01e-47 |
| Low | CSI | BMI | 375 | -0.020 | 0.702 | 0.789 |
| Low | CSI | Total medication burden | 465 | 0.273 | 2.04e-9 | 3.81e-9 |
| Low | CSI | CNS medication burden | 465 | 0.280 | 7.47e-10 | 1.49e-9 |
| Low | BDI | MPQ | 456 | 0.479 | 1.66e-27 | 5.80e-27 |
| Low | BDI | SF-36 Vitality | 450 | -0.603 | 6.70e-46 | 3.75e-45 |
| Low | BDI | BMI | 384 | 0.016 | 0.757 | 0.789 |
| Low | BDI | Total medication burden | 474 | 0.265 | 4.39e-9 | 7.23e-9 |
| Low | BDI | CNS medication burden | 474 | 0.264 | 5.22e-9 | 8.12e-9 |
| Low | MPQ | SF-36 Vitality | 445 | -0.418 | 2.73e-20 | 5.88e-20 |
| Low | MPQ | BMI | 368 | 0.190 | 2.48e-4 | 3.16e-4 |
| Low | MPQ | Total medication burden | 457 | 0.221 | 1.76e-6 | 2.34e-6 |
| Low | MPQ | CNS medication burden | 457 | 0.230 | 7.01e-7 | 9.81e-7 |
| Low | SF-36 Vitality | BMI | 366 | 0.025 | 0.637 | 0.775 |
| Low | SF-36 Vitality | Total medication burden | 451 | -0.263 | 1.37e-8 | 2.02e-8 |
| Low | SF-36 Vitality | CNS medication burden | 451 | -0.276 | 2.41e-9 | 4.23e-9 |
| Low | BMI | Total medication burden | 419 | 0.015 | 0.756 | 0.789 |
| Low | BMI | CNS medication burden | 419 | -0.002 | 0.965 | 0.965 |
| Low | Total medication burden | CNS medication burden | 520 | 0.948 | 8.31e-260 | 2.33e-258 |
| High | FIQR | CSI | 164 | 0.425 | 1.41e-8 | 6.59e-8 |
| High | FIQR | BDI | 167 | 0.385 | 2.77e-7 | 1.11e-6 |
| High | FIQR | MPQ | 163 | 0.549 | 3.20e-14 | 4.49e-13 |
| High | FIQR | SF-36 Vitality | 163 | -0.253 | 0.001 | 0.003 |
| High | FIQR | BMI | 163 | 0.112 | 0.153 | 0.269 |
| High | FIQR | Total medication burden | 175 | 0.043 | 0.569 | 0.637 |
| High | FIQR | CNS medication burden | 175 | 0.008 | 0.917 | 0.951 |
| High | CSI | BDI | 164 | 0.491 | 2.61e-11 | 2.43e-10 |
| High | CSI | MPQ | 163 | 0.461 | 6.15e-10 | 4.30e-9 |
| High | CSI | SF-36 Vitality | 163 | -0.320 | 3.08e-5 | 1.08e-4 |
| High | CSI | BMI | 153 | 0.096 | 0.238 | 0.317 |
| High | CSI | Total medication burden | 164 | 0.119 | 0.128 | 0.255 |
| High | CSI | CNS medication burden | 164 | 0.116 | 0.138 | 0.258 |
| High | BDI | MPQ | 163 | 0.290 | 1.70e-4 | 5.28e-4 |
| High | BDI | SF-36 Vitality | 163 | -0.439 | 4.69e-9 | 2.63e-8 |
| High | BDI | BMI | 156 | 0.136 | 0.089 | 0.200 |
| High | BDI | Total medication burden | 167 | 0.108 | 0.163 | 0.269 |
| High | BDI | CNS medication burden | 167 | 0.098 | 0.208 | 0.291 |
| High | MPQ | SF-36 Vitality | 163 | -0.101 | 0.198 | 0.291 |
| High | MPQ | BMI | 152 | 0.093 | 0.254 | 0.323 |
| High | MPQ | Total medication burden | 163 | -0.046 | 0.559 | 0.637 |
| High | MPQ | CNS medication burden | 163 | -0.072 | 0.360 | 0.439 |
| High | SF-36 Vitality | BMI | 152 | -0.137 | 0.093 | 0.200 |
| High | SF-36 Vitality | Total medication burden | 163 | 0.025 | 0.753 | 0.811 |
| High | SF-36 Vitality | CNS medication burden | 163 | 0.002 | 0.983 | 0.983 |
| High | BMI | Total medication burden | 163 | 0.144 | 0.067 | 0.169 |
| High | BMI | CNS medication burden | 163 | 0.099 | 0.208 | 0.291 |
| High | Total medication burden | CNS medication burden | 175 | 0.945 | 1.09e-85 | 3.06e-84 |

Note. Correlations were calculated separately within the low-impact (FIQR <62) and high-impact (FIQR ≥62) groups. Pairwise complete observations were used, so N varies across correlations. P values were adjusted within each phenotype using the Benjamini-Hochberg false discovery rate procedure. FIQR, Revised Fibromyalgia Impact Questionnaire; CSI, Central Sensitization Inventory; BDI, Beck Depression Inventory; MPQ, McGill Pain Questionnaire; BH, Benjamini-Hochberg.

**Supplementary Table S3A. Regression model diagnostics.**

| **Model** | **N** | **R²** | **Adjusted R²** | **Residual SD** | **Max VIF** | **Mean VIF** | **Max Cook's D** | **Cook's D >4/n, n** | **Max leverage** | **Leverage >2p/n, n** | **Max \|studentized residual\|** | **\|Studentized residual\| >3, n** | **Shapiro-Wilk P** | **Breusch-Pagan P** |
| --- | --- | --- | --- | --- | --- | --- | --- | --- | --- | --- | --- | --- | --- | --- |
| CNS medication burden model | 507 | 0.352 | 0.342 | 7.42 | 2.780 | 1.622 | 0.0319 | 30 | 0.0555 | 25 | 2.906 | 0 | 0.083 | 5.60e-6 |
| Total medication burden model | 507 | 0.354 | 0.344 | 7.40 | 2.763 | 1.622 | 0.0312 | 33 | 0.0562 | 28 | 2.901 | 0 | 0.097 | 6.95e-6 |

Note. Cook's distance was evaluated against 4/n; leverage was evaluated against 2p/n, where p includes the intercept. No observation had an externally studentized residual with an absolute value greater than 3. Shapiro-Wilk results did not indicate significant departure from residual normality; Breusch-Pagan tests indicated heteroscedasticity in both models. Robust standard errors or heteroscedasticity-consistent sensitivity analyses should therefore be considered if these models are retained.

**Supplementary Table S3B. Variance inflation factors and tolerance.**

| **Model** | **Predictor** | **VIF** | **Tolerance** |
| --- | --- | --- | --- |
| CNS medication burden model | Male sex (vs female) | 1.050 | 0.952 |
| CNS medication burden model | CSI score | 2.780 | 0.360 |
| CNS medication burden model | BDI score | 2.137 | 0.468 |
| CNS medication burden model | MPQ score | 2.138 | 0.468 |
| CNS medication burden model | BMI | 1.102 | 0.907 |
| CNS medication burden model | Age | 1.085 | 0.921 |
| CNS medication burden model | CNS medication burden | 1.059 | 0.945 |
| Total medication burden model | Male sex (vs female) | 1.050 | 0.952 |
| Total medication burden model | CSI score | 2.763 | 0.362 |
| Total medication burden model | BDI score | 2.146 | 0.466 |
| Total medication burden model | MPQ score | 2.138 | 0.468 |
| Total medication burden model | BMI | 1.106 | 0.904 |
| Total medication burden model | Age | 1.085 | 0.922 |
| Total medication burden model | Total medication burden | 1.069 | 0.935 |

Note. All VIF values were below 3, indicating no evidence of problematic multicollinearity.

**Supplementary Table S4. Medication classification framework.**

| **Medication class** | **Agents represented** | **Centrally acting** | **Included in total burden** | **Included in CNS burden** | **Classification rationale** |
| --- | --- | --- | --- | --- | --- |
| Tricyclic antidepressants | Amitriptyline, desipramine, doxepin, imipramine, nortriptyline | Yes | Yes | Yes | Central pain modulation, sleep, and affective regulation |
| Gabapentinoid | Gabapentin | Yes | Yes | Yes | Central modulation of sensory processing and pain |
| Gabapentinoid | Pregabalin | Yes | Yes | Yes | Central modulation of sensory processing and pain |
| SNRI | Duloxetine | Yes | Yes | Yes | Descending pain modulation and affective regulation |
| SNRI | Venlafaxine | Yes | Yes | Yes | Descending pain modulation and affective regulation |
| SSRI | Fluoxetine | Yes | Yes | Yes | Affective and centrally mediated symptom regulation |
| SNRI | Milnacipran | Yes | Yes | Yes | Descending pain modulation |
| Anticonvulsant | Topiramate | Yes | Yes | Yes | Central neuromodulatory effects |
| Atypical antidepressant | Bupropion | Yes | Yes | Yes | Central psychotropic effect |
| Serotonergic antidepressant | Trazodone | Yes | Yes | Yes | Sleep and affective regulation |
| NSAIDs | Celecoxib, naproxen, ibuprofen, meloxicam, indomethacin | No | Yes | No | Predominantly peripheral anti-inflammatory/analgesic action |
| Skeletal muscle relaxant | Cyclobenzaprine | Yes | Yes | Yes | Centrally acting muscle relaxant |
| Skeletal muscle relaxant | Metaxalone | Yes | Yes | Yes | Centrally acting muscle relaxant |
| Skeletal muscle relaxant | Tizanidine | Yes | Yes | Yes | Centrally acting alpha-2 agonist |
| Skeletal muscle relaxant | Carisoprodol | Yes | Yes | Yes | Centrally acting muscle relaxant |
| Skeletal muscle relaxant | Baclofen | Yes | Yes | Yes | Central GABA-B agonist |
| Centrally acting analgesic | Tramadol | Yes | Yes | Yes | Opioid and monoaminergic central analgesic effects |
| None of the listed medications | None of the above | No | No | No | Indicator only; does not contribute to medication counts |

Note. Total medication burden was defined as the count of listed medications reported by each participant. CNS medication burden was defined as the count of agents classified as centrally acting on the basis of predominant effects on pain modulation, affective regulation, sleep, sensory processing, or nociceptive amplification. The aggregate CNS category quantifies cumulative centrally acting pharmacologic exposure and does not imply identical mechanisms or therapeutic effects across classes.

**Supplementary Table S5. Full multivariable linear regression outputs for SF-36 Physical Component Summary models.**

| **Model** | **Predictor** | **N** | **β** | **Standardized β** | **SE** | **95% CI** | **t** | **P** | **R²** | **Adjusted R²** | **Model F** | **Model P** |
| --- | --- | --- | --- | --- | --- | --- | --- | --- | --- | --- | --- | --- |
| CNS medication burden model | Male sex (vs female) | 507 | 0.570 | — | 1.122 | -1.635 to 2.774 | 0.51 | 0.612 | 0.352 | 0.342 | 38.64 | 2.62e-43 |
| CNS medication burden model | CSI score | 507 | -0.230 | -0.444 | 0.032 | -0.292 to -0.168 | -7.24 | 1.68e-12 | 0.352 | 0.342 | 38.64 | 2.62e-43 |
| CNS medication burden model | BDI score | 507 | 0.131 | 0.147 | 0.045 | 0.042 to 0.220 | 2.90 | 0.004 | 0.352 | 0.342 | 38.64 | 2.62e-43 |
| CNS medication burden model | MPQ score | 507 | -0.042 | -0.216 | 0.010 | -0.061 to -0.023 | -4.30 | 2.02e-5 | 0.352 | 0.342 | 38.64 | 2.62e-43 |
| CNS medication burden model | BMI | 507 | -0.175 | -0.161 | 0.039 | -0.251 to -0.099 | -4.53 | 7.56e-6 | 0.352 | 0.342 | 38.64 | 2.62e-43 |
| CNS medication burden model | Age | 507 | -0.073 | -0.109 | 0.024 | -0.120 to -0.026 | -3.04 | 0.003 | 0.352 | 0.342 | 38.64 | 2.62e-43 |
| CNS medication burden model | CNS medication burden | 507 | -0.628 | -0.068 | 0.322 | -1.261 to 0.004 | -1.95 | 0.052 | 0.352 | 0.342 | 38.64 | 2.62e-43 |
| Total medication burden model | Male sex (vs female) | 507 | 0.530 | — | 1.121 | -1.671 to 2.732 | 0.47 | 0.636 | 0.354 | 0.344 | 38.99 | 1.22e-43 |
| Total medication burden model | CSI score | 507 | -0.231 | -0.446 | 0.032 | -0.293 to -0.169 | -7.30 | 1.13e-12 | 0.354 | 0.344 | 38.99 | 1.22e-43 |
| Total medication burden model | BDI score | 507 | 0.135 | 0.151 | 0.045 | 0.046 to 0.224 | 2.98 | 0.003 | 0.354 | 0.344 | 38.99 | 1.22e-43 |
| Total medication burden model | MPQ score | 507 | -0.042 | -0.215 | 0.010 | -0.061 to -0.023 | -4.29 | 2.16e-5 | 0.354 | 0.344 | 38.99 | 1.22e-43 |
| Total medication burden model | BMI | 507 | -0.172 | -0.157 | 0.039 | -0.248 to -0.096 | -4.44 | 1.13e-5 | 0.354 | 0.344 | 38.99 | 1.22e-43 |
| Total medication burden model | Age | 507 | -0.073 | -0.110 | 0.024 | -0.120 to -0.026 | -3.06 | 0.002 | 0.354 | 0.344 | 38.99 | 1.22e-43 |
| Total medication burden model | Total medication burden | 507 | -0.660 | -0.080 | 0.284 | -1.218 to -0.101 | -2.32 | 0.021 | 0.354 | 0.344 | 38.99 | 1.22e-43 |

Note. The dependent variable was the SF-36 Physical Component Summary score. Separate models evaluated CNS medication burden and total medication burden. Both models included CSI, BDI, MPQ, BMI, age, and sex. β denotes the unstandardized regression coefficient. Standardized β is not shown for sex because it is categorical. CI, confidence interval; CSI, Central Sensitization Inventory; BDI, Beck Depression Inventory; MPQ, McGill Pain Questionnaire; BMI, body mass index.
